# Effects of different mixed sowing ratios of oat and forage pea on forage yield and quality in alpine region

**DOI:** 10.3389/fpls.2026.1803106

**Published:** 2026-08-18

**Authors:** Wenting Ma, Zeliang Ju, Lianxue Duan, Jing Pan, Xiaolong Ma, Zhifeng Jia, Xiang Ma

**Affiliations:** Key Laboratory of Superior Forage Germplasm in the Qinghai-Tibetan Plateau, Qinghai Academy of Animal Science and Veterinary Medicine, Qinghai University, Xining, China

**Keywords:** feeding distance value, forage pea, mixture ratio, nutritional quality, oat, output

## Abstract

To optimize planting patterns for artificial grassland in the Guinan area, this study systematically compared the forage productivity, nutritional quality, and feeding value of four oat (*Avena sativa* L.)–forage pea (*Pisum sativum* L.) mixture ratios (8:2, 7:3, 6:4, and 5:5) with two monoculture controls (oat ‘Qinghai 444’ and forage pea ‘Qingjian No.1’). The 7:3 mixture achieved significantly greater hay yield and crude protein (CP) content than both monocultures and all other mixture ratios (*P* < 0.05), indicating a synergistic interaction between the cereal and legume components. Feeding value indices—including relative feed value (RFV), relative forage quality (RFQ), total digestible nutrients (TDN), and net energy for lactation (NEL)—were likewise maximized under this ratio. Furthermore, entropy-weighted TOPSIS comprehensive evaluation confirmed that the 7:3 ratio attained the highest integrated score for both yield performance and nutritional quality, significantly outperforming traditional monoculture systems. These results demonstrate that a 7:3 seeding ratio of oat to forage pea represents an optimal strategy for artificial grassland establishment in the Guinan area, simultaneously enhancing forage productivity and nutritional value while providing a practical reference for sustainable mixed forage systems in the region.

## 1 Introduction

Forage production in alpine regions is constrained by short growing seasons, low temperatures, and poor soil fertility, under which monocultures of oat (*Avena sativa* L.) and forage pea (*Pisum sativum* L.) often exhibit suboptimal performance (Zhang et al., 2014). While oat monoculture produces high biomass, its crude protein content is typically low and its productivity is heavily dependent on soil nitrogen supply; in nitrogen-deficient alpine soils, this reliance leads to reduced yield and nutritional value. In contrast, forage pea monoculture can fix atmospheric nitrogen via rhizobial symbiosis; however, under harsh alpine conditions, it is characterized by poor standability, low total biomass, and weak stems prone to lodging, which complicates harvesting and can cause yield loss and mold-induced quality deterioration. To address these limitations, cereal–legume intercropping has gained considerable attention, and the oat–forage pea mixture has been widely studied because it capitalizes on complementary traits between the two species (Chai et al., 2022; Li et al., 2023; Zhu et al., 2023). Specifically, oat provides structural support that reduces forage pea lodging, whereas forage pea contributes biological nitrogen fixation; a portion of the fixed nitrogen is transferred to the oat through root exudates and residue decomposition, thereby improving system-level nitrogen use efficiency. This interspecific facilitation enhances overall forage yield and nutritional quality, enabling the mixture to outperform monocultures of either species.

Oat–forage pea mixtures can efficiently utilize spatial, water, light, and soil resources, and under appropriate mixing ratios, they achieve higher forage yield and more comprehensive nutritional quality than monocultures (Kızıl Aydemir and Kızılşimşek, 2019; Ma et al., 2025). Oat is a high-quality annual gramineous forage widely cultivated for its high yield, stress resistance, and excellent palatability (Liu et al., 2023). Its crude protein content is more than twice that of silage corn and grain straw, whereas its fiber content is lower, ensuring good palatability and improving nutrient absorption and digestibility in livestock (Niu et al., 2020). Nevertheless, continuous oat monoculture is unsustainable because it leads to declining yield and quality, aggravated pest and disease pressure, and soil degradation. Forage pea has high crude protein content, good palatability, and high digestibility; however, as a climbing plant, it is prone to lodging under monoculture, resulting in low land-use efficiency, reduced yield, and high labor costs at harvest (Kostrzewska et al., 2022). Consequently, oat–forage pea mixed sowing has emerged as an effective strategy to improve both yield and quality by exploiting interspecific differences in light, water, and nutrient use (Lithourgidis et al., 2006). Previous studies have examined the production performance of mixed grasslands from multiple perspectives, including sowing combinations, ratios, densities, and cultivation measures such as fertilization and ridge planting (Kang et al., 2023; Luo et al., 2023; Tamiru et al., 2023; Yao et al., 2024). A 7:3 oat–forage pea ratio has been reported to increase crude protein content by 2.47 percentage points compared with oat monoculture, substantially improving nutritional quality (Feng et al., 2025). Similarly, this ratio has been found to significantly increase fresh grass and hay yields by 10.15 t·hm^-^² and 2.61 t·hm^-^², respectively, relative to oat monoculture (Xue et al., 2025). Selecting an appropriate mixing ratio is therefore critical to ensure that the synergistic effect between oat and forage pea exceeds their competitive interaction, thereby maximizing the advantages of mixed sowing (Yan et al., 2025).

Research on oat–forage pea mixtures in alpine regions has expanded recently, and the benefits of such systems are well documented (Bo et al., 2022). However, most existing studies on grass–legume mixtures in alpine regions rely on single-season or single-year observations, leaving the long-term stability of species interactions poorly understood. Second, previous efforts to identify optimal mixture ratios have typically depended on unidimensional indicators—such as yield or crude protein alone—rather than integrating productivity, nutritional quality, and feeding value into a unified evaluation framework. Third, the specific response of locally adapted oat and forage pea varieties to mixing ratios under the harsh climatic conditions of the Guinan area remains poorly quantified. We therefore hypothesized that a 7:3 oat–forage pea seeding ratio would achieve a synergistic optimum among yield, nutritional quality, and feeding value through interspecific nitrogen facilitation and structural complementarity, and that this advantage would remain stable across consecutive growing seasons. To test this hypothesis, the present study established a two-year fixed-site field experiment with the following objectives: (1) to systematically compare agronomic traits, forage yield, and nutritional quality among four mixture ratios (8:2, 7:3, 6:4, and 5:5) and two monoculture controls; (2) to identify the optimal mixture ratio and evaluate its inter-annual stability; and (3) to conduct a comprehensive, multi-criteria evaluation using the entropy-weighted TOPSIS method to provide a quantitative decision-making basis for sustainable artificial grassland establishment in the Guinan alpine region.

## 2 Materials and methods

### 2.1 General situation of the test site

The experimental site was located in Taxiu Township (100.75°E, 35.57°N), Guinan County, Hainan Tibetan Autonomous Prefecture, Qinghai Province, at an average altitude of 3,700 m. The field experiment was conducted during the 2023 and 2024 growing seasons. Mean annual air temperature was 3.0 °C in 2023 and 3.7 °C in 2024, with total annual precipitation of 632.6 mm and 555.9 mm, respectively. The region is characterized by a cold and dry continental plateau climate with no absolute frost-free period, thin air, intense solar radiation, and marked diurnal temperature variation. The forage growing season spans approximately 150 days, and the dominant soil types are chestnut soil and chernozem.

### 2.2 Test materials

The oat variety tested was’ Qinghai 444 ‘. This oat variety exhibits strong adaptability and early maturity in alpine regions. The leguminous forage pea variety is ‘ Qingjian No.1 ‘. This pea variety has a high yield.; they were provided by the Grassland Institute of Qinghai Academy of Animal Husbandry and Veterinary Sciences.

### 2.3 Experimental design

The experiment employed a randomized block design comprising six sowing treatments (Y, S, T1, T2, T3, T4; see **Table 1** for details), each replicated three times for a total of 18 plots. Each plot measured 20 m² (4 m × 5 m) with a row spacing of 25 cm, an inter-plot spacing of 0.5 m, and a sowing depth of 3–5 cm. Diammonium phosphate (150 kg ha^-^²) and urea (75 kg ha^-^²) were applied as a base fertilizer prior to sowing. The plants received two sessions of manual weeding between the tillering and jointing stages. No additional fertilization or irrigation was provided throughout the growing season. Aligned with regional climate and established planting practices, we conducted the experiment annually on May 29. For the mixed sowing, both species were sown together in the same row. The seeding rates for oats and forage peas per row were determined as a specific percentage of their monoculture sowing rates. Taking oat + forage pea 8:2 as an example, oat sowing rate = oat monoculture sowing rate 165 kg·hm ^−2^ × 80% = 132 kg·hm ^−2^, forage pea sowing rate = forage pea monoculture sowing rate 120 kg·hm ^−2^ × 20% = 24 kg·hm ^−2^, the specific sowing rate is shown in **Table 1**. This study carried out a comprehensive evaluation of different grass-legume mixed sowing ratios in the Guinan area during the 2023 and 2024 growing seasons, and it selected the best mixed sowing ratio.

**Table 1:** Different mixing ratios and sowing rates.

Treatment	Mixing ratio	Seeding rate (kg·hm^-2^)	
		Avena sativa	Pisum sativa
Y	1:0	165.00	–
S	0:1	–	120.00
T1	8:2	132.00	24.00
T2	7:3	115.50	36.00
T3	6:4	99.00	48.00
T4	5:5	82.50	60.00

-Represents not sown the material.

### 2.4 Indicators and methods of determination

#### 2.4.1 Agronomic traits

Plant height and stem diameter: At the oat milk stage, we randomly selected ten representative plants each of oats and forage peas from each plot, choosing individuals with uniform growth and an absence of pests and diseases. We measured the absolute height from the ground to the highest point using a tape measure (accuracy: 1 cm) and recorded it as the plant height. We then measured the diameter at the second basal internode of the oat stems using a vernier caliper (Zhang et al., 2020) and recorded this value as the stem diameter.

Flag leaf length, width, and area: Measurements were taken at the oat milk ripening stage. Ten plants with consistent growth, representative characteristics, and no pest or disease symptoms were randomly selected from each plot for sampling. The maximum length and maximum width of the flag leaf of the main stem were measured by a tape measure, which were recorded as the length of the flag leaf (L) and the width of the flag leaf (W), respectively. The flag leaf area (AL) was determined by the formula established by Liang et al (Liang et al., 2018):


AL=R×L×W

In the formula, R is the oat leaf area correction system (0.8317).

#### 2.4.2 Yield determination

The fresh and hay yield and the ratio of fresh to dry: Oat milk ripening period sampling, each plot to the side line, cutting 1m^2^, weighing the fresh weight of forage, then put into the air-dried bag, bring back to the laboratory, in 105 °C oven to kill green 30 min, 65 °C drying to constant weight, weighing and calculating the hay yield, calculate the ratio of fresh to dry (Favre et al., 2019).

#### 2.4.3 Nutritional quality

Quality determination: The hay samples were crushed and dried using a grinder. Three samples were randomly selected from the mixed grass samples to determine crude ash (Ash), crude protein (CP), crude fat (EE), neutral detergent fiber (NDF), and acid detergent fiber (ADF). The nutritional components were analyzed using the following methods: ash by dry ashing, crude protein (CP) by the Kjeldahl method, ether extract (EE) with a FOSS Soxtec TM 8000 fat analyzer, and neutral and acid detergent fibers (NDF, ADF) by the Van Soest method. At the same time, the feeding value indexes of forage were calculated, which were relative feeding value (RFV), relative forage quality (RFQ), total digestible nutrients (TDN) (Gao et al., 2021; Liu et al., 2016), and net energy for milk production (NEL).


DMI(%) = 120/NDF

DDM(%) = 88.9−0.779ADF

RFV = DMI × DDM/1.29

TDN = 82.38 − 0.7515ADF

RFQ = TDN × DMI/1.23

NL=[1.085−(0.0124×ADF)]×9.29

In the formula, NDF and ADF are neutral detergent fiber content and acid detergent fiber content, respectively; DMI is dry matter intake; DDM is digestible dry matter; 1.29 is the parameter value for forage DMI × DDM; RFV is relative feeding value; RFQ is relative forage quality; TDN is total digestible nutrient; NEL is net energy of milk production.

### 2.5 Data analysis

Data management and visualization were performed using Microsoft Office Excel 2021. Statistical analysis was carried out with SPSS 27.0, employing one-way ANOVA with a significance level of *P* < 0.05. Data for all measured indicators are presented as the mean ± standard deviation.

This study utilized the entropy weight-TOPSIS method for a comprehensive evaluation of the forage production performance under different treatments, aiming to select the most suitable grass-legume mixed sowing ratio in the Guinan area. The following preparations were conducted prior to the analysis. 1.Constructing the evaluation index system and matrix; 2. Determining the index weights via the entropy weight method; and 3. Constructing a weighted normalized matrix and an ideal solution. 4. Calculate the Euclidean distance. 5. Calculate the closeness and sort. This method combines the entropy weight method and the TOPSIS method. The evaluation process involved two main steps. First, the entropy weight method determined the index weights. This was followed by the TOPSIS method, which ranked the comprehensive performance of all treatments based on their Euclidean distances to the positive and negative ideal solutions. The greater the relative proximity value, the better the comprehensive performance of a treatment, and vice versa.

## 3 Results

### 3.1 Comparison of forage agronomic traits under different treatments

In 2023, both oat and pea plant heights were significantly greater under the T2 treatment than under all other treatments (P < 0.05). The lowest oat plant height was recorded under T1, whereas the lowest pea plant height was observed under S. No significant differences in stem diameter were detected among treatments (P > 0.05). Oat flag leaf width was greatest under T1, with no significant differences among intercropping treatments (P > 0.05). Both oat flag leaf length and flag leaf area were maximized under T2; these parameters were significantly greater than those under T3, T4, and S (P < 0.05), but did not differ significantly from those under T1.

In 2024, both oat and pea plant heights under T2 were significantly higher than those under other treatments (P < 0.05). The lowest oat plant height was observed under T3, while the lowest pea plant height was again recorded under S. Stem diameter displayed a pattern consistent with that of 2023. Oat flag leaf length was greatest under T2, differing significantly only from T4 (P < 0.05), with no significant differences from T1, T3, or S (P > 0.05). Oat flag leaf width and flag leaf area followed patterns consistent with those observed in 2023.

Averaged across the two years, both oat and pea plant heights were greatest under T2; the lowest oat plant height was observed under T3, whereas the lowest pea plant height remained under S. Stem diameter was relatively consistent across treatments. Oat flag leaf length was greatest under T2, while flag leaf width was maximized under T1. Oat flag leaf area under T2 was significantly greater than that under all other treatments (P < 0.05) (**Table 2**).

**Table 2:** Effects of different mixed sowing ratios on agronomic traits of forage grass.

Years	Treatment	Oat plant height/cm	Legume plant height/cm	Stem thick/mm	Flag length/cm	Flag leaf width/cm	Leaf area/cm^2^
2023	Y	154.08 ± 2.46ab	–	5.48 ± 0.43a	25.48 ± 3.80b	2.03 ± 0.23b	42.87 ± 7.58c
	S	–	82.26 ± 3.76d	–	–	–	–
	T1	148.16 ± 2.61c	107.30 ± 2.93c	5.93 ± 0.57a	28.42 ± 4.31ab	2.40 ± 0.20a	56.55 ± 8.35ab
	T2	159.08 ± 4.67a	120.60 ± 6.47a	5.95 ± 0.57a	32.58 ± 2.57a	2.38 ± 0.19a	64.62 ± 9.98a
	T3	152.52 ± 4.65bc	109.84 ± 5.51bc	5.32 ± 0.81a	26.62 ± 3.97b	2.30 ± 0.30ab	50.70 ± 8.34bc
	T4	152.42 ± 4.73bc	113.52 ± 5.24b	5.40 ± 0.84a	25.54 ± 3.78b	2.33 ± 0.12ab	49.57 ± 8.45bc
2024	Y	138.80 ± 5.76d	–	5.48 ± 0.94a	24.20 ± 4.31ab	2.26 ± 0.24b	45.24 ± 6.95d
	S	–	85.81 ± 4.02d	–	–	–	–
	T1	145.40 ± 4.88c	106.38 ± 3.26c	5.46 ± 0.87a	24.50 ± 3.33ab	2.80 ± 0.23a	56.82 ± 7.20b
	T2	160.20 ± 4.66a	120.85 ± 2.32a	6.38 ± 0.91a	29.50 ± 1.87a	2.76 ± 0.39a	67.41 ± 7.34a
	T3	135.60 ± 4.39d	111.52 ± 4.67b	5.70 ± 0.83a	25.38 ± 2.33ab	2.64 ± 0.23ab	55.70 ± 6.56bc
	T4	152.20 ± 4.32b	114.66 ± 5.76b	6.37 ± 0.89a	23.50 ± 1.22b	2.42 ± 0.19ab	47.39 ± 5.53cd
mean value	Y	146.44 ± 9.07c	–	5.48 ± 0.69a	24.84 ± 3.89b	2.15 ± 0.25b	44.06 ± 6.97d
	S	–	84.03 ± 4.12d	–	–	–	–
	T1	146.78 ± 3.96bc	106.84 ± 2.96c	5.71 ± 0.74a	26.46 ± 4.18b	2.60 ± 0.29a	56.69 ± 7.35b
	T2	159.64 ± 4.44a	120.73 ± 4.58a	6.17 ± 0.75a	31.04 ± 2.67a	2.57 ± 0.35a	66.01 ± 8.39a
	T3	144.06 ± 9.88c	110.68 ± 4.90b	5.51 ± 0.80a	26.00 ± 3.14b	2.47 ± 0.31a	53.20 ± 7.55bc
	T4	152.31 ± 4.28b	114.09 ± 5.23b	5.89 ± 0.97a	24.52 ± 2.96b	2.38 ± 0.16ab	48.48 ± 6.83cd

### 3.2 Comparison of forage yield under different treatments

In 2023, fresh and dry herbage yields were highest under T2 and lowest under Y, with T2 exceeding Y by 17.66 and 4.44 t·hm^-^², respectively. No significant differences in the fresh-to-dry ratio were detected among treatments (P > 0.05); the maximum and minimum values were recorded under T4 and Y, respectively.

In 2024, fresh herbage yield was highest under S, whereas dry herbage yield was greatest under T2, though no significant differences were observed for the latter across treatments (P > 0.05). Both fresh and dry herbage yields were lowest under Y. Relative to Y, fresh herbage yield under S was 22.72 t·hm^-^² higher, and dry herbage yield under T2 was 3.15 t·hm^-^² higher. The fresh-to-dry ratio was highest under S; however, it did not differ significantly from those under T2, T3, or T4 (P > 0.05), and was lowest under Y.

Averaged across the two years, fresh and dry herbage yields were highest under T2 and lowest under Y. Compared with Y, all intercropping treatments increased both fresh and dry herbage yields. The fresh-to-dry ratio was relatively high under S and low under Y (**Table 3**).

**Table 3:** Effects of different mixed sowing ratios on forage yield.

Years	Treatment	Fresh yield/(t·hm^−2^)	Dry yield/(t·hm^−2^)	Fresh-dry ratio
2023	Y	34.89 ± 3.83c	14.63 ± 1.21c	2.41 ± 0.42a
	S	43.02 ± 4.77abc	17.08 ± 1.08b	2.52 ± 0.17a
	T1	42.07 ± 7.95bc	15.42 ± 0.85c	2.72 ± 0.48a
	T2	52.55 ± 2.97a	19.07 ± 0.72a	2.75 ± 0.06a
	T3	48.89 ± 7.12ab	17.97 ± 0.47ab	2.72 ± 0.43a
	T4	43.99 ± 3.30abc	15.27 ± 0.53c	2.89 ± 0.29a
2024	Y	37.55 ± 2.85c	16.59 ± 2.21a	2.29 ± 0.35b
	S	60.27 ± 2.13a	18.13 ± 0.64a	3.33 ± 0.18a
	T1	43.17 ± 4.74c	17.46 ± 1.02a	2.47 ± 0.16b
	T2	55.53 ± 5.12ab	19.74 ± 1.43a	2.83 ± 0.41ab
	T3	50.67 ± 5.12b	18.71 ± 3.69a	2.81 ± 0.75ab
	T4	43.33 ± 1.85c	16.92 ± 1.50a	2.57 ± 0.16ab
mean value	Y	36.22 ± 3.35d	15.61 ± 1.92d	2.35 ± 0.35b
	S	51.64 ± 10.00a	17.61 ± 0.98abc	2.92 ± 0.47a
	T1	42.62 ± 5.89cd	16.44 ± 1.40bcd	2.60 ± 0.35ab
	T2	54.04 ± 4.08a	19.41 ± 1.08a	2.79 ± 0.27ab
	T3	49.78 ± 5.63ab	18.34 ± 2.39ab	2.76 ± 0.55ab
	T4	43.66 ± 2.42bc	16.09 ± 1.35cd	2.73 ± 0.27ab

### 3.3 Comparison of nutritional quality of forage under different treatments

In 2023, Ash content was significantly greater under T4 than under all other treatments (P < 0.05), with the lowest value recorded under Y. Crude protein (CP) content exceeded 7% across all treatments, and was significantly higher under S than under any other treatment (P < 0.05); the lowest value was observed under Y. Ether extract (EE) content ranged from 3.01% to 3.90%, peaking under T3 and reaching a minimum of 3.01% under T4. Neutral detergent fiber (NDF) content varied between 48.63% and 60.55%, with the lowest value under T2 and the highest under T1, which significantly exceeded that under all other treatments (P < 0.05). Acid detergent fiber (ADF) content ranged from 30.84% to 37.27%, with the lowest value under S and the highest under Y, which was significantly greater than that under all other treatments (P < 0.05).

In 2024, Ash content remained significantly higher under T4 than under all other treatments (P < 0.05), with Y again recording the lowest value. All treatments had CP contents above 6%; S again produced the highest CP content, significantly exceeding that under all other treatments (P < 0.05), with Y again having the lowest. EE content ranged from 3.11% to 3.70%, with the highest value under T2 and the lowest under T4. NDF content varied between 47.56% and 56.16%, with the lowest value under S and the highest under T1, which significantly exceeded that under all treatments except T4 (P < 0.05). ADF content ranged from 31.58% to 37.52%, with the lowest value under S and the highest under T1, which was significantly greater than that under all treatments except T4 (P < 0.05).

Averaged across the two years, Ash content was highest under T4, significantly greater than that under all other treatments (P < 0.05). Mean CP content exceeded 7% across all treatments, with the highest value under S, significantly exceeding that under all other treatments (P < 0.05), and the lowest under Y. Mean EE content was highest under T3. T1 recorded the highest mean NDF and ADF contents, whereas S had the lowest values for both parameters (**Table 4**).

**Table 4:** Effects of different mixed sowing ratios on nutritional quality of forage grass.

Years	Treatment	Ash	CP	EE	NDF	ADF
2023	Y	5.90 ± 0.19d	7.33 ± 0.90e	3.67 ± 0.27ab	59.47 ± 0.56b	37.27 ± 0.63a
	S	6.73 ± 0.22bc	17.61 ± 0.74a	3.18 ± 0.24c	51.94 ± 0.42e	30.84 ± 0.78d
	T1	6.97 ± 0.25b	8.53 ± 1.01de	3.31 ± 0.19bc	60.55 ± 0.76a	35.37 ± 0.47b
	T2	6.56 ± 0.37bc	11.28 ± 0.57b	3.73 ± 0.26ab	48.63 ± 0.47f	32.50 ± 0.77c
	T3	6.42 ± 0.40c	10.04 ± 0.88bc	3.90 ± 0.22a	58.31 ± 0.50c	34.53 ± 0.70b
	T4	7.65 ± 0.28a	9.62 ± 0.55cd	3.01 ± 0.24c	57.26 ± 0.53d	35.06 ± 0.82b
2024	Y	5.55 ± 0.38d	6.68 ± 0.29d	3.59 ± 0.18a	52.89 ± 1.31bc	34.85 ± 1.39b
	S	6.24 ± 0.29bc	18.12 ± 0.80a	3.16 ± 0.11b	47.56 ± 1.64d	31.58 ± 1.43d
	T1	6.71 ± 0.30b	8.80 ± 0.34c	3.20 ± 0.12b	56.16 ± 0.94a	37.52 ± 0.90a
	T2	6.52 ± 0.32bc	10.52 ± 0.59b	3.70 ± 0.23a	51.30 ± 1.06c	32.91 ± 0.60cd
	T3	6.02 ± 0.23cd	10.39 ± 0.74b	3.66 ± 0.20a	52.48 ± 1.06c	34.25 ± 1.01bc
	T4	8.73 ± 0.38a	9.30 ± 0.47c	3.11 ± 0.17b	54.54 ± 1.11ab	36.05 ± 1.11ab
mean value	Y	5.73 ± 0.33d	7.01 ± 0.70e	3.63 ± 0.21a	56.18 ± 3.72a	36.06 ± 1.64a
	S	6.49 ± 0.35bc	17.86 ± 0.75a	3.17 ± 0.17b	49.75 ± 2.62b	31.21 ± 1.10d
	T1	6.84 ± 0.29b	8.67 ± 0.69d	3.26 ± 0.15b	58.36 ± 2.52a	36.45 ± 1.34a
	T2	6.54 ± 0.31bc	10.90 ± 0.68b	3.72 ± 0.22a	49.97 ± 1.64b	32.71 ± 0.66c
	T3	6.22 ± 0.36c	10.22 ± 0.75b	3.78 ± 0.23a	55.39 ± 3.28a	34.39 ± 0.79b
	T4	8.19 ± 0.66a	9.46 ± 0.49c	3.06 ± 0.19b	55.90 ± 1.68a	35.56 ± 1.02ab

### 3.4 Comparison of the forage value of different treatments

'In 2023, RFV was highest under T2 and lowest under Y, with T2 significantly exceeding all other treatments (P < 0.05). RFQ followed the same pattern, being highest under T2 and lowest under Y, with significant differences between T2 and the other treatments (P < 0.05). TDN was highest under S and lowest under Y, with S significantly outperforming all other treatments (P < 0.05). Similarly, NEL was highest under S and lowest under Y, with significant differences (P < 0.05). In 2024, RFV was highest under S and lowest under T1, with significant differences between S and all other treatments (P < 0.05). RFQ was also highest under S and lowest under T1, with S significantly exceeding the other treatments (P < 0.05). TDN was highest under S and lowest under T1, and NEL was highest under S and lowest under T1. Averaged across the two years, S achieved the highest values for RFV, RFQ, TDN, and NEL, whereas T1 recorded the lowest values for all four indices. Compared with S, T1 reduced RFV, RFQ, TDN, and NEL by 20%, 28%, 6.6%, and 9.3%, respectively (**Table 5**).

**Table 5:** Effects of different mixed sowing ratios on the feeding value of forage grass.

Years	Treatment	RFV	RFQ	TDN	NEL
2023	Y	93.65 ± 0.72d	115.56 ± 1.41d	54.37 ± 0.47d	5.79 ± 0.07d
	S	116.19 ± 0.83b	159.53 ± 1.62b	59.20 ± 0.59a	6.53 ± 0.09a
	T1	94.26 ± 1.61d	116.77 ± 3.15d	55.80 ± 0.35c	6.00 ± 0.05c
	T2	121.63 ± 2.34a	170.13 ± 4.57a	57.96 ± 0.58b	6.34 ± 0.09b
	T3	98.92 ± 1.69c	125.84 ± 3.29c	56.43 ± 0.53c	6.10 ± 0.08c
	T4	100.05 ± 1.52c	128.05 ± 2.96c	56.03 ± 0.61c	6.04 ± 0.09c
2024	Y	108.69 ± 4.54bc	144.89 ± 8.85bc	56.19 ± 1.04cd	6.06 ± 0.16c
	S	125.91 ± 6.54a	178.48 ± 8.78a	58.65 ± 1.07a	6.44 ± 0.16a
	T1	98.86 ± 2.78d	125.73 ± 5.43d	54.18 ± 0.67d	5.76 ± 0.10d
	T2	114.73 ± 2.35b	156.68 ± 4.58b	57.65 ± 0.45ab	6.29 ± 0.07ab
	T3	110.34 ± 3.62bc	148.11 ± 7.05bc	56.64 ± 0.76bc	6.13 ± 0.12bc
	T4	103.77 ± 3.54cd	135.30 ± 6.90cd	55.29 ± 0.83cd	5.93 ± 0.13cd
mean value	Y	101.17 ± 8.74bc	130.23 ± 17.00bc	55.28 ± 1.23d	5.93 ± 0.19d
	S	121.05 ± 6.76a	169.01 ± 11.82a	58.92 ± 0.83a	6.48 ± 0.13a
	T1	96.56 ± 3.24c	121.25 ± 6.31c	54.99 ± 1.01d	5.88 ± 0.15d
	T2	118.18 ± 4.32a	163.41 ± 8.43a	57.80 ± 0.50b	6.31 ± 0.08b
	T3	104.63 ± 6.75b	136.98 ± 13.15b	56.53 ± 0.59c	6.12 ± 0.09c
	T4	101.91 ± 3.17bc	131.68 ± 6.19bc	55.66 ± 0.77cd	5.98 ± 0.12cd

### 3.5 Evaluation of the correlation between forage growth indexes and nutritional quality and feeding value indexes of different mixed sowing ratios

**Figure 1:**
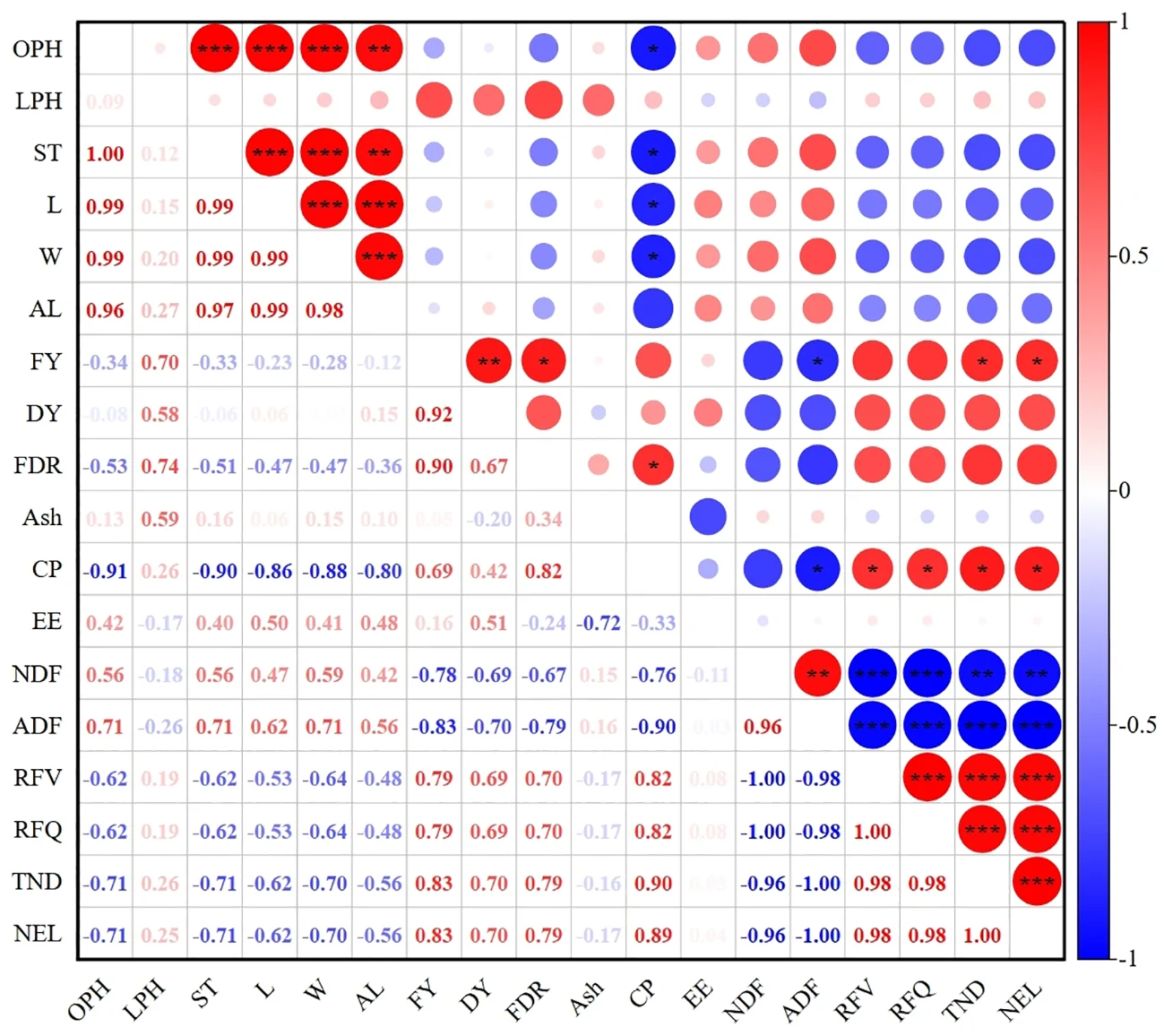
Correlation analysis of forage yield, growth index, nutritional quality, and feeding value of different mixed sowing ratios. OPH represents oat plant height, LPH represents leguminous plant height, ST represents stem diameter, L represents flag leaf length, W represents flag leaf width, AL represents flag leaf area, FY represents fresh grass yield, DY represents hay yield, FDR represents fresh to dry ratio, Ash represents crude ash, CP represents crude protein, EE represents crude fat, NDF represents neutral detergent fiber, ADF represents acid detergent fiber, RFV represents relative feeding value, RFQ represents relative forage quality, TDN represents total digestible nutrients, NEL represents milk net energy. The chromaticity of the color in the figure represents the correlation coefficient (R), where red represents a positive correlation and blue represents a negative correlation. *, **, *** represented significant *P* < 0.05, *P* < 0.01 and *P* < 0.001, respectively.

The correlation analysis results of forage growth indexes, nutritional quality and feeding value indexes of different mixed sowing ratios showed that (**Figure 1**), the plant height of oat was significantly positively correlated with stem diameter, flag leaf length and flag leaf width(*P* < 0.001), and significantly positively correlated with flag leaf area (*P* < 0.01); the stem diameter was significantly positively correlated with flag leaf length and width (*P* < 0.001), and significantly positively correlated with flag leaf area (*P* < 0.01); flag leaf length was significantly positively correlated with flag leaf width and flag leaf area (*P* < 0.001), flag leaf width was significantly positively correlated with flag leaf area (*P* < 0.001), the plant height, stem diameter, flag leaf length and flag leaf width of oat were significantly negatively correlated with crude protein content (*P* < 0.05); fresh grass yield was significantly positively correlated with hay yield (*P* < 0.01), and significantly positively correlated with fresh-dry ratio, total digestible nutrients, and net milk production (*P* < 0.05), but significantly negatively correlated with acid detergent fiber (*P* < 0.05). There was a significant positive correlation between fresh-dry ratio and crude protein content (*P* < 0.05). Crude protein content was positively correlated with relative feeding value, relative forage quality, total digestible nutrients and net energy of milk production (*P* < 0.05) but negatively correlated with acid detergent fiber (*P* < 0.05). Neutral detergent fiber was significantly positively correlated with acid detergent fiber (*P* < 0.01). Still, it significantly negatively correlated with relative feeding value and relative forage quality (*P* < 0.001), and significantly negatively correlated with total digestible nutrients and net milk production (*P* < 0.01). Acid detergent fiber (ADF) was strongly and negatively correlated with key feeding value parameters—including relative feeding value, relative forage quality, total digestible nutrients, and net energy for lactation (P < 0.001). Relative feeding value was significantly positively correlated with relative forage quality, total digestible nutrients, and net milk production (*P* < 0.001), and relative feeding quality was significantly positively correlated with total digestible nutrients, and net milk production (*P* < 0.001). There was a significant positive correlation between total digestible nutrients and net milk production (*P* < 0.001). In summary, oat plant height is the key factor to determine the growth index of forage grass, and neutral detergent fiber and acid detergent fiber are the key factors to determine the nutritional value of forage grass.

### 3.6 Comprehensive evaluation of the forage productivity of different treatments

**Figure 2:**
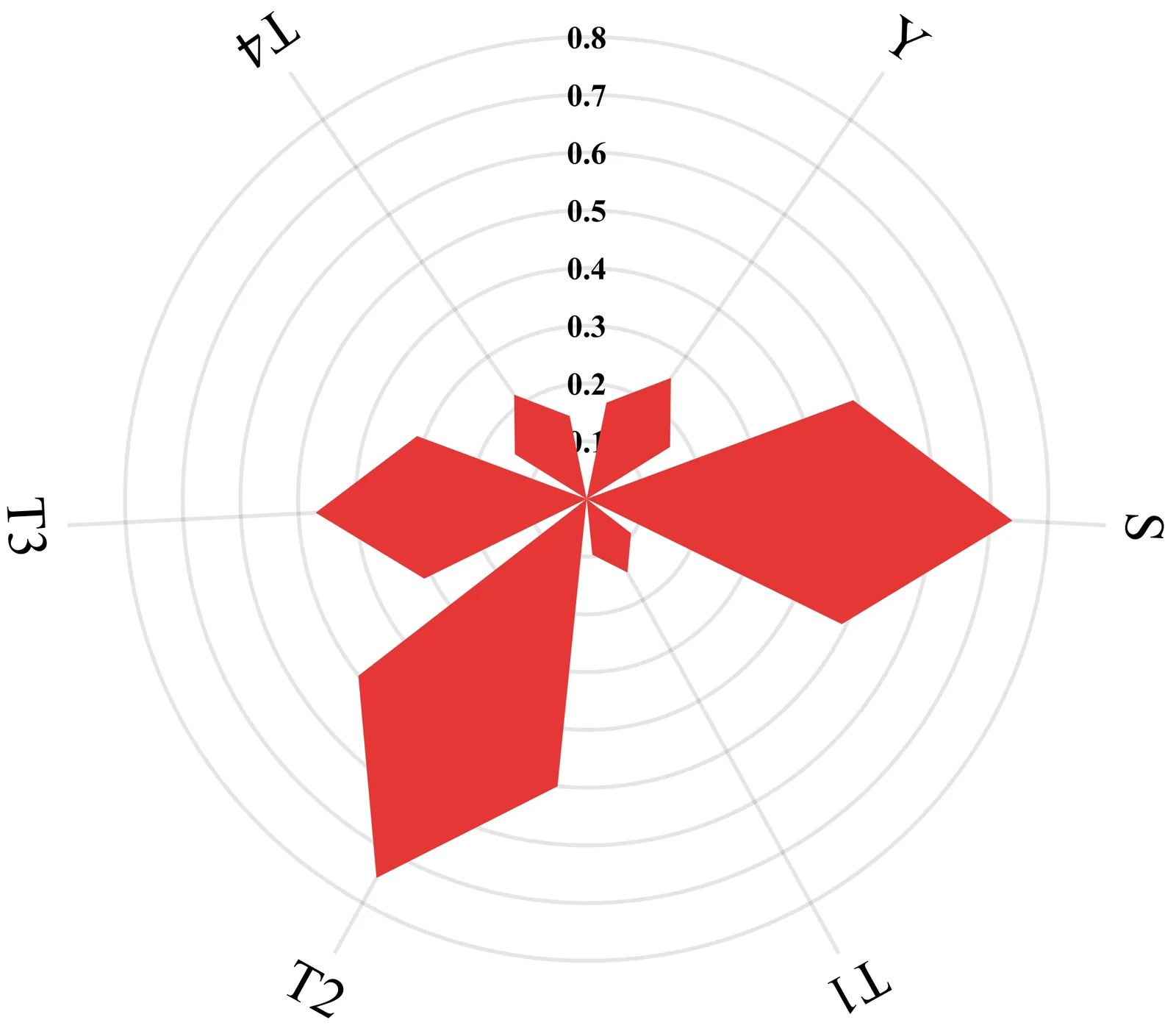
Comprehensive evaluation of forage grass under different treatments.

This study employed the entropy weight-TOPSIS method to comprehensively evaluate and rank the relative forage production performance of the various treatments, using the two-year average data (**Figure 2**). The results showed that the order of forage production capacity of different therapies was: T2 > S > T3 > Y > T4 > T1, and the order of closeness was: 0.751,0.739,0.470,0.255,0.220,0.146. The T2 treatment (oat + forage pea 7:3) demonstrated optimal performance; the T1 treatment (oat + forage pea 8:2), on the other hand, yielded the poorest results.

## 4 Discussions

### 4.1 Effects of mixing ratios on forage yield and growth characteristics

Yield traits of cereal–legume mixed forage can indirectly reflect the growth characteristics and productive performance of forage crops. Previous studies have demonstrated that morphological indices such as plant height, stem diameter, and leaf area in grass–legume mixtures are closely associated with yield; however, these parameters cannot be simultaneously optimized, necessitating comprehensive regulation through mixed-seeding ratios (Chen et al., 2020; Ko, 2019; Wu et al., 2022). Appropriate mixed-seeding combinations and ratios can enhance light and nutrient use efficiency through interspecific facilitation and competition, thereby forming stable community structures and achieving stable or increased yields (Tracy and Sanderson, 2004). Among these traits, plant height serves as a critical morphological indicator for assessing forage growth and development. Across the six mixed-seeding treatments evaluated in this study, the oat and ‘Qingjian 1’ pea mixture at a 7:3 ratio produced the greatest plant heights for both oat (159.64 cm) and forage pea (120.73 cm), representing increases of 8.3% and 30% relative to sole-cropped oat and sole-cropped pea, respectively. Mixed cropping enhanced forage plant height, a finding consistent with the results reported by Zhao et al. (2024). The increased oat plant height may be attributed to nitrogen supplied by pea promoting nitrogen uptake and vertical growth, whereas the increased pea plant height likely results from structural support provided by oat, facilitating climbing and improving light and temperature use efficiency. These observations indicate that rational mixed cropping can synergistically improve forage plant height (OKCU et al., 2025). Stem diameter is an important factor ensuring high forage yield, as individual plants can increase biomass per plant and enhance lodging resistance through accelerated lateral stem growth, thereby influencing forage yield (Munkaila and Islam, 2025). In the present study, mixed-seeding ratios had no significant effect on oat stem diameter; however, the 7:3 oat-to-forage pea treatment produced the thickest stems, and all mixed-seeding treatments exhibited greater stem diameters than sole-cropped oat, with increases ranging from 4.5% to 11.1%. This may be explained by nitrogen provided by forage pea promoting cellulose and lignin synthesis in oat, thereby optimizing stem structure and ultimately increasing stem diameter (Wu et al., 2025a). Leaf quality and leaf area can indirectly reflect photosynthetic capacity and, consequently, the productive performance of forage crops under different treatments. In this experiment, the 7:3 oat-to-forage pea treatment exhibited a significantly greater oat flag leaf area than all other treatments, with the most favorable leaf characteristics, indicating the highest light use efficiency and superior productive performance under this treatment (Kang et al., 2023; Wu et al., 2025b; Xie et al., 2025).

Different cereal–legume mixing ratios exert varying effects on forage yield and quality (Silva et al., 2022). Research conducted in the Yushu region has shown that a triticale–common vetch mixture at a 0.7:0.3 ratio achieved superior forage yield and quality compared with other mixing ratios (Tong et al., 2025). Similarly, a study by Qian (2023) in the arid loess plateau of eastern Gansu demonstrated that a 70:30 cereal–legume mixing ratio maximized hay yield. In the present study, the oat and ‘Qingjian 1’ pea mixture at a 7:3 ratio produced fresh and dry herbage yields of 54.04 and 19.41 t·ha^-^¹, respectively, exceeding those of other mixed-seeding treatments by 7.9%–21.1% and 5.5%–17.1%. The optimal mixing ratio for maximizing fresh and dry weights observed here aligns with findings reported by others (Hou et al., 2025). Under this 7:3 ratio, oat constituted the principal yield component; its erect stems formed a three-dimensional canopy with strong light-capture capacity (Acar et al., 2017). Meanwhile, the moderate density of forage pea allowed it to climb the oat stems, thereby avoiding ground contact and rot, while continuously supplying nitrogen to oat through rhizobial nitrogen fixation (Ma, 2019). This promoted increased oat tillering and biomass accumulation, maximizing interspecific facilitation and minimizing interspecific competition, ultimately achieving the highest hay yield across all treatments (Ma et al., 2015; Wei et al.,2024).

### 4.2 Effects of mixed-seeding ratios on forage nutritional quality and feeding value

Different mixing ratios exert varying effects on forage quality enhancement (Komac et al., 2014). Contents of crude protein (CP), ether extract (EE), neutral detergent fiber (NDF), and acid detergent fiber (ADF) are critical metrics for assessing forage nutritional quality (Wang et al., 2024a). In this study, sole-cropped pea exhibited the highest CP content. Following oat–pea mixed cropping, overall CP content increased markedly and was significantly greater than that under sole-cropped oat, which may be attributed to the inherently high CP concentrations characteristic of leguminous forages. As an important energy-yielding constituent, EE content also influences forage palatability (Guo et al., 2025). The 6:4 oat-to-’Qingjian 1’ pea treatment produced the highest EE content, followed by the 7:3 ratio, indicating that specific mixing ratios can effectively improve forage quality (Liu et al., 2025).

NDF and ADF contents affect livestock feed intake and digestibility (Wang et al., 2022). NDF determines satiety and is negatively correlated with voluntary feed intake, whereas ADF reflects the less digestible fiber fraction and is negatively associated with digestibility (Wang et al., 2024b); consequently, higher ADF content indicates lower digestibility and reduced energy availability. In the present study, NDF contents of all mixed treatments except the 9:1 oat-to-forage pea ratio were lower than that of sole-cropped oat. The 7:3 oat-to-forage pea treatment exhibited the lowest NDF and ADF contents. Notably, the 7:3 ratio produced lower NDF and ADF than the 8:2 ratio, whereas the 8:2 ratio (T1) exhibited elevated NDF. One possible hypothesis is that this pattern may be hypothetically linked to the competition–facilitation balance.

In the 8:2 mixture (T1), oat was overwhelmingly dominant, with a seeding rate as high as 132 kg·ha^-^¹ (approximately 80%) and intense intraspecific competition due to high plant density. It is possible that the limited pea component was insufficient to promote oat leaf expansion through nitrogen transfer, resulting in a higher proportion of stems in the harvested biomass and, consequently, elevated overall fiber concentrations (Gadisa et al., 2023). By contrast, under the 7:3 ratio (T2), the 30% forage pea component may have supplied adequate nitrogen to support oat leaf expansion. As shown in **Table 2**, T2 exhibited the greatest flag leaf area (66.01 cm²), and the 70% oat component may have provided sufficient structural support for the pea. Hypothetically, if the canopy leaf-to-stem ratio under this treatment was higher than that under other treatments, fiber concentration may have been relatively diluted due to an increased leaf proportion. Although the mechanism of reducing fiber content through increased leaf area has been documented in grass–legume mixed-cropping studies (Brink et al., 2015), given that the actual botanical composition and leaf-to-stem ratio were not determined in this study, the above explanations represent reasonable inferences based solely on the available data and remain to be further validated.

In the present study, the progressive increase in NDF and ADF from the 7:3 to the 6:4 and 5:5 oat-to-’Qingjian 1’ forage pea ratios may reflect a deterioration in sward structure attributable to intensified interspecific competition. A plausible hypothesis is that once the pea proportion exceeded the optimal threshold, competition between the two species for light, water, and nitrogen may have intensified, suppressing oat growth, as evidenced by marked reductions in plant height and flag leaf area (Koc et al., 2013). Concurrently, the reduced oat proportion may have compromised structural support for the climbing forage pea, potentially increasing the incidence of stem entanglement, lodging, and the incorporation of basal stems into the harvested biomass, thereby possibly reducing the leaf-to-stem ratio (Temesgen, 2025). The decline in leaf proportion relative to stem fraction would be expected to elevate ADF concentrations. Notably, the ascending gradient in fiber content across the 5:5, 6:4, and 7:3 treatments (5:5 > 6:4 > 7:3) is highly consistent with the hypothesized severity of community structural degradation.

The crude protein (CP) concentration of the 7:3 oat-to-’Qingjian 1’ pea mixture (10.90%) was greater than that of the 5:5 mixture (9.46%), despite the latter theoretically containing a higher legume proportion. This apparent paradox may be tentatively explained by the following hypothesis: under the 7:3 ratio, effective nitrogen transfer from forage pea to oat may have fostered interspecific facilitation between the two species, promoting greater leaf expansion in oat and potentially increasing the leaf proportion relative to total plant biomass. By contrast, an alternative hypothesis is that the 5:5 mixture may have been dominated by intense interspecific competition, which could have suppressed forage pea growth and constrained nitrogen fixation efficiency. Furthermore, T2 (the 7:3 treatment) exhibited the greatest leaf area; because leaves are inherently richer in protein than stems, a higher leaf-to-stem ratio—if present—could potentially account for the higher overall CP concentration (Mulcahy and Stuart, 2024). In the 5:5 mixture, however, competitive stress and an elevated lodging tendency may have increased the stem fraction in the harvested biomass, thereby hypothetically diluting the protein concentration relative to the 7:3 treatment.

Among the mixed-seeding treatments evaluated in this study, the 7:3 oat-to-’Qingjian 1’ forage pea mixture exhibited the highest values for RFV, RFQ, TDN, and NEL, and consequently attained the greatest feeding value; by contrast, the 8:2 oat-to-forage pea mixture recorded the lowest values for all four indices and was characterized by the poorest feeding value. Accordingly, a correlation analysis was conducted between forage nutritional components and feeding value (**Figure 1**). The analysis revealed that NDF and ADF contents were the primary determinants of feeding value, both of which were negatively correlated with the evaluated feeding value indices.

It must be emphasized that the mechanistic interpretations presented above regarding the effects of mixed-seeding ratios on forage quality parameters remain hypothetical. In the present experiment, the actual botanical composition of the harvested forage (i.e., the relative proportions of oat and forage pea biomass), together with the leaf-to-stem ratio, lodging severity, and nitrogen transfer efficiency, were not directly determined. Consequently, the reliability of the feeding value indices (RFV, RFQ, TDN, and NEL) calculated on this basis is necessarily constrained. Future studies are required to validate these interpretations through direct measurement of species-specific biomass allocation, morphological traits, and symbiotic nitrogen fixation at harvest.

### 4.3 Comprehensive evaluation of forage adaptability under different mixed-seeding ratios

Existing comprehensive evaluation studies of cereal–legume mixed forage systems have been largely confined to forage yield and conventional nutritional quality metrics, yielding unidimensional assessment frameworks that inadequately reflect the actual feeding potential of forage crops. The present study expanded upon this approach by integrating feeding value parameters—including RFV, RFQ, TDN, and NEL—into a multidimensional evaluation system, enabling precise identification of the optimal oat–forage pea mixing ratio for cultivated pastures in the Guinan region. An entropy-weighted TOPSIS model was employed to conduct the comprehensive assessment, thereby enhancing the robustness and objectivity of the derived conclusions (Zhang et al., 2015). Relative closeness coefficients indicated that the 7:3 oat-to-’Qingjian 1’ forage pea mixture, sole-cropped ‘Qingjian 1’ pea, and the 6:4 oat-to-forage pea mixture achieved the three highest composite scores, with the 7:3 ratio exhibiting the most favorable overall performance. This pattern is consistent with the findings of Qian (2023) regarding cereal–legume intercropping in alpine regions. In contrast, the 8:2 oat-to-forage pea mixture yielded the poorest comprehensive evaluation, attributable primarily to the incorporation of multiple feeding value indices into the assessment framework; the significantly lower RFV, RFQ, TDN, and NEL values of this treatment substantially depressed its overall score. Collectively, the entropy-weighted TOPSIS evaluation demonstrates that the 7:3 oat-to-’Qingjian 1’ forage pea ratio achieves simultaneous optimization of dry herbage yield, nutritional quality, and feeding value, objectively establishing this mixing ratio as the ideal cultivation pattern for annual sown pastures in the Guinan region.

Although this study provides a multidimensional assessment of oat–forage pea mixed-seeding ratios for the Guinan region, several limitations merit acknowledgment. First, the two-year, fixed-site experiment lacked automatic weather stations, precluding precise quantification of climatic variables that mediate interspecific competition and facilitation. Future research should therefore incorporate automatic meteorological monitoring and adopt multi-year, multi-site designs to evaluate the stability of mixed-cropping systems under contrasting climatic years. Second, the absence of pre-experimental soil baseline information represents not merely a data gap, but a methodological limitation. Without baseline soil nutrient data, this further constrains the interpretation of treatment effects in this experiment. We are unable to determine whether the observed differences in forage yield and quality among treatments were genuinely induced by the mixed-seeding ratios themselves or influenced by inherent spatial heterogeneity in soil fertility across the experimental plots. Therefore, future studies should conduct comprehensive soil nutrient measurements at both the initiation and termination of the experiment, incorporating baseline soil nutrients into the analytical framework to improve the accuracy of treatment effect evaluation. Finally, expanding the evaluation to multiple agro-ecological zones across the Qinghai–Tibet Plateau would enhance the geographical representativeness and practical applicability of the optimal mixing ratio identified herein. .

## 5 Conclusion

In the mixed sowing experiment of oat and forage pea in the Guinan area, the comprehensive evaluation of six different mixed sowing ratios showed that the fresh forage yield, hay yield, nutritional quality, and feeding value of the oat + forage pea ‘Qingjian No.1’ 7:3 treatment were the best under the tested conditions. The entropy weight-TOPSIS results indicated that the adaptability ranking of the tested combinations was 7:3 > 0:1 > 6:4 > 1:0 > 5:5 > 8:2. These findings suggest that a 7:3 seeding ratio represents a promising strategy for artificial grassland establishment at the experimental site, simultaneously enhancing forage productivity and nutritional value. However, we acknowledge that this conclusion is based on a single fixed-site experiment conducted over two growing seasons. Further multi-location validation across diverse alpine pastoral areas, economic feasibility analyses, and long-term assessments of mixture stability and species interactions are needed before broad agronomic recommendations can be made for the entire Guinan region or the Qinghai–Tibet.

## Data Availability

The original contributions presented in the study are included in the article/supplementary material. Further inquiries can be directed to the corresponding authors.
